# Intraoperative low-dose dopamine is associated with worse survival in patients with hepatocellular carcinoma: A propensity score matching analysis

**DOI:** 10.3389/fonc.2022.947172

**Published:** 2022-08-25

**Authors:** Yan Wang, Ruifeng Xue, Wei Xing, Qiang Li, Liba Gei, Fang Yan, Dongmei Mai, Weian Zeng, Yan Yan, Dongtai Chen

**Affiliations:** ^1^ Department of Anesthesiology, Sun Yat-sen University Cancer Center, State Key Laboratory of Oncology in South China, Collaborative Innovation Center for Cancer Medicine, Guangzhou, China; ^2^ Department of Anesthesiology, Inner Mongolia Autonomous Region Cancer Hospital, Affiliated People’s Hospital of Inner Mongolia Medical University, Hohhot, China; ^3^ Department of Anesthesiology, Huizhou Municipal Central Hospital, Huizhou, China

**Keywords:** hepatocellular carcinoma, hepatectomy, low-dose dopamine, recurrence, survival

## Abstract

**Background:**

Dopamine is widely used in patients during surgery. We evaluated the association between intraoperative low-dose dopamine administration and recurrence-free survival (RFS) and overall survival (OS) in patients with hepatocellular carcinoma (HCC).

**Methods:**

Consecutive patients with nonmetastatic HCC who underwent radical hepatectomy were enrolled between 2008 and 2010. Univariate and multivariate logistic regression analyses were used to evaluate the prognostic factors for RFS and OS. Survival outcomes were evaluated using Kaplan–Meier analyses with the log-rank test. A one-to-one propensity score matching (PSM) analysis was performed to reduce confounding bias.

**Results:**

A total of 805 HCC patients, including 699 patients who did not receive dopamine consumption and 106 patients who received low-dose dopamine during the operation, were retrospectively analyzed. The patients who were assigned low-dose dopamine had worse RFS (*p* = 0.009) and OS (*p* = 0.041) than those who did not receive dopamine. Multivariate regression analysis showed that the intraoperative administration of low-dose dopamine was an independent unfavorable predictor for RFS (*p* = 0.004) but not for OS (*p* = 0.059). After PSM, the low-dose dopamine-treated group still had significantly poorer RFS (*p* = 0.003) and OS (*p* = 0.002). When stratified by time of recurrence, patients with low-dose dopamine use had a significantly greater chance of recurrence within 2 years (*p* = 0.007) but not after 2 years (*p* = 0.186).

**Conclusions:**

Intraoperative low-dose dopamine use has a negative impact on RFS and OS in HCC patients who have undergone radical hepatectomy. Further prospective studies are required to assess the effects of low-dose dopamine on surgical outcomes in HCC patients.

## Introduction

Hepatocellular carcinoma (HCC) is one of the leading causes of cancer death worldwide, accounting for 75-85% of primary liver cancer (1). Despite the improvement of surgical treatment and targeted therapy, the outcomes of HCC patients remain unfavorable because of the high rates of cancer recurrence and mortality (2–4).

Currently, hepatic resection is the mainstay of HCC treatment. However, intraoperative anesthetic management has a critical effect on HCC patients. During liver resection, maintaining low central venous pressure (CVP) is considered an important management aspect of hepatic parenchyma dissection to reduce intraoperative blood loss (5, 6). Low CVP can be achieved by restricting fluid input, increasing urine output, clamping the inferior vena cava and so on (7, 8). Nevertheless, lower CVP measures frequently lead to some complications, such as vital organ perfusion insufficiency and hemodynamic instability.

Dopamine, as an endogenous catecholamine, affects renal perfusion and cardiovascular control in a dose-dependent manner. Anesthesiologists usually adjust the dosage of dopamine due to the vital signs of patients during surgical operations. In clinical practice, low-dose dopamine, as a renal dose, is often applied less than 3 μg/kg/min to increase renal blood flow and urine volume (9), which is an anesthetic technique to maximize renal perfusion. This might be one of the reasons that low-dose dopamine use is widely administered in hepatic surgery.

The influence of anesthetic approaches on cancer patients is complex. Growing evidence from animal and human studies has revealed that the different types of anesthetic procedures can affect the tumor progression and survival outcomes in patients with malignancies (10–12). Our recent study found that dopamine promotes the proliferation, migration and invasion of HCC cell lines *in vitro* (13). However, few clinical trials have been performed to investigate the impact of dopamine use on survival outcomes in cancer patients due to the specific tumor microenvironment during surgery. Therefore, we conducted a retrospective cohort study of patients with HCC undergoing open hepatectomy to explore the association between intraoperative low-dose dopamine administration and the survival outcomes of the patients.

## Materials and methods

### Patients and study design

We retrospectively selected a total of 952 consecutive patients with newly-diagnosed nonmetastatic HCC at Sun Yat-sen University Cancer Center (SYSUCC) between January 2008 and December 2010. The inclusion criteria were as follows: (1) patients who underwent open radical hepatectomy; (2) patients who had postoperative tumor-free margins; and (3) patients who had complete clinicopathological and follow-up data. The exclusion criteria were as follows: (1) patients who had another primary malignancy before the diagnosis of HCC; (2) patients who received preoperative therapy; (3) patients who had severe preoperative physical conditions, such as Child–Pugh class C liver function, renal dysfunction and severe cardiovascular disease; and (4) patients who admitted surgical intensive care unit after surgery. The selected clinicopathological data were as follow: (1) patient characteristics before hepatectomy (sex, age, American Society of Anesthesiologists (ASA) physical status, HBsAg, cirrhosis, serum alpha-fetoprotein (AFP), gamma glutamyl transferase (GGT), alanine aminotransferase (ALT), aspartate aminotransferase (AST), bilirubin, albumin and creatinine); (2) malignant tumor factors (tumor size, tumor number, satellite nodules and vascular invasion); (3) administration of intraoperative low-dose dopamine (1-2 μg/kg/min); (4) clinical parameters during surgery (intraoperative fluid infusion, urine output, norepinephrine use, blood loss and duration of surgery) and within one week after operation (postoperative AFP, ALT, AST, bilirubin and creatinine) and (5) the time of tumor recurrence and death. This study conformed to the Declaration of Helsinki and was approved by the Institutional Ethics Committees of the SYSUCC (approval number: B2022-065-01). Owing to the nature of the retrospective study, the requirement for written informed consent was waived by the Institutional Review Board.

### Surgical treatment and follow-up

Open radical hepatectomy was performed or supervised by two consultant hepatic surgeons on the same treatment team. The surgical procedure was determined based on the patient tumor number, tumor size, liver function and physical status. All patients with HCC received regular follow-up every 3 months for the first 2 years after surgery and then every 6 months thereafter. Each follow-up consisted of blood tests and imaging examinations, including serum AFP, liver function tests, and at least one abdominal imaging scan, such as ultrasound, computed tomography (CT) or magnetic resonance imaging (MRI). Annually, chest CT was screened as a standard procedure. Enrolled patients were followed up until tumor recurrence or death or until December 2021. HCC recurrence was classified into early or late recurrence by using 2 years as the cut-off (14–16) and was diagnosed by one of the following criteria: (a) liver tissue pathological diagnosis; and (b) typical lesion appearances in abdominal enhanced-contrast CT or MRI (hypervascularity enhanced on the arterial phase and washout on the portal venous phase) (2). The sites of recurrence included intrahepatic and extrahepatic recurrence. Recurrence-free survival (RFS) was measured as the interval between the date of initial hepatectomy and the date of recurrence, death from disease or the last follow-up. Overall survival (OS) was measured as the survival time from the date of initial hepatectomy to the date of death or the last follow-up.

### Statistical analysis

Categorical data were analyzed with the chi-square test or Fisher’s exact test, as appropriate. Continuous data were analyzed with the *t*-test or Wilcoxon rank-sum test. RFS and OS in two cohorts comprising intraoperative low-dose dopamine and without low-dose dopamine were assessed by Kaplan–Meier analysis and were compared using a log-rank test. Univariate and multivariate logistic regression analyses were used to evaluate the prognostic factors for RFS and OS. To reduce selection bias and balance variables, a 1:1 matched cohort using propensity score matching (PSM) analysis was performed. All statistical tests were two-sided, and *P* value less than 0.05 were considered statistically significant. Except for the Kaplan-Meier curves, which were analyzed by the website statistical tool (http://www.bioinformatics.com.cn/), other statistical analyses were performed using SPSS software, version 22 (IBM Corporation, Armonk, NY, USA).

## Results

### Patient characteristics

**Figure 1:**
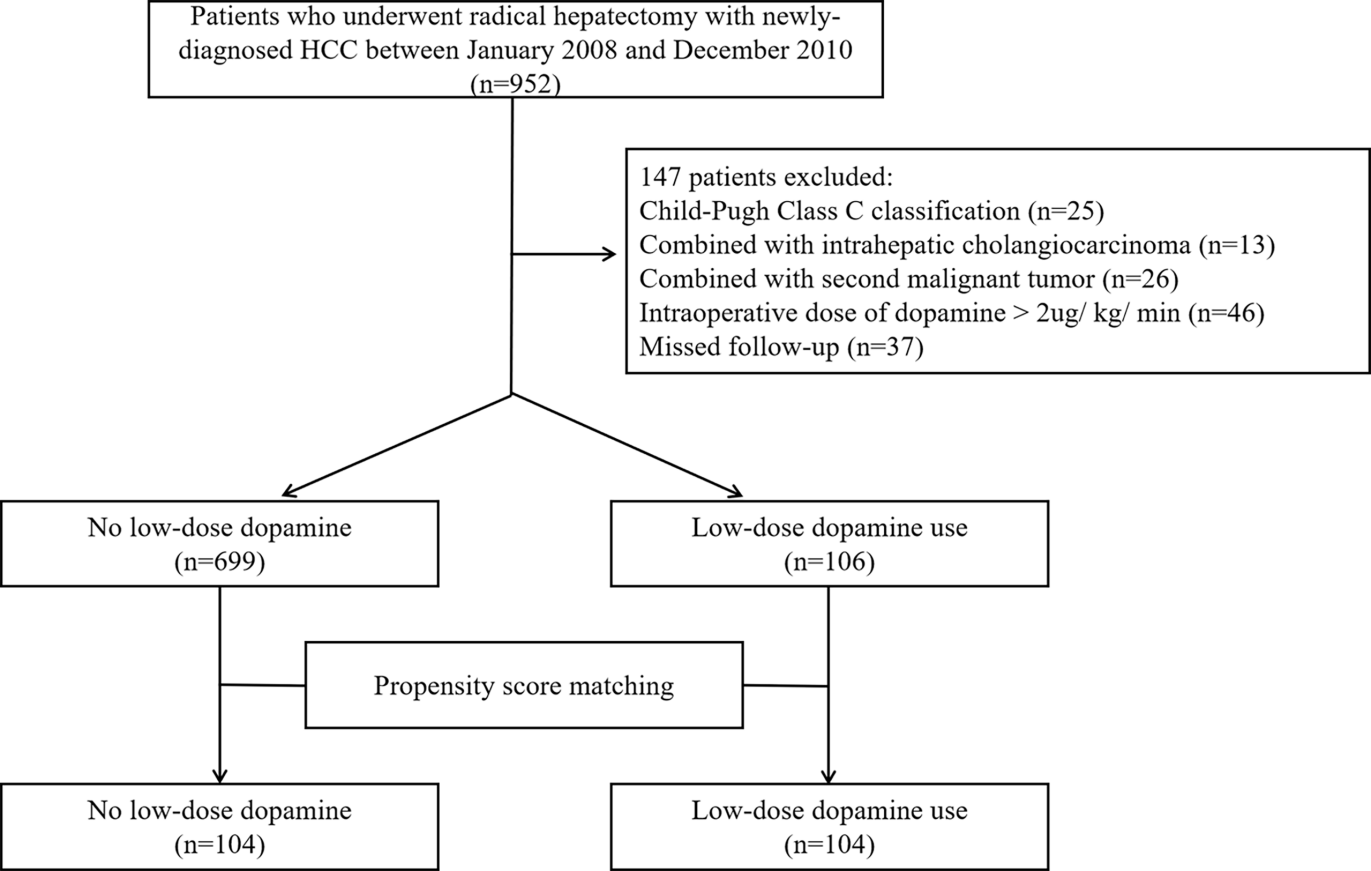
Flowchart of the patient selection.

From the initial group of 952 HCC patients, 147 were excluded according to the criteria. Ultimately, a total of 805 patients were enrolled in this study, including 699 patients who did not consume dopamine and 106 patients who received low-dose dopamine during radical hepatectomy (**Figure 1**). The baseline characteristics of the original cohort are summarized in **Table 1**. Before PSM, low-dose dopamine use was associated with a higher grade of ASA physical status (*p* = 0.036), larger tumor size (*p* = 0.032), lower AFP level (*p* = 0.015) and lower ALT level (*p* = 0.008). There were no significant differences between the two groups in terms of sex, age, HBsAg, cirrhosis, Child–Pugh classification, tumor number, satellite nodules, vascular invasion, AST level, total bilirubin level, direct bilirubin level, albumin level, or creatinine (all *p* > 0.05). In terms of intraoperative and postoperative clinical characteristics, there were no significant differences in all variables between the two groups, including intraoperative fluid infusion, urine output, norepinephrine use, blood loss and duration of operation, postoperative AFP, ALT, AST, bilirubin and creatinine (all *p* > 0.05) (**Supplementary Table 1**).

**Table 1:** Patient characteristics with low-dose dopamine administered before and after propensity score matching.

Characteristics	Before matching			After matching		
	Low-dose dopamine			Low-dose dopamine		
	No (n=699)	Yes (n=106)	*P*-value	No (n=104)	Yes (n=104)	*P*-value
Sex						
Female	73 (10.4%)	8 (7.5%)		11 (10.6%)	8 (7.7%)	
Male	626 (89.6%)	98 (92.5%)	0.356	93 (89.4%)	96 (92.3%)	0.470
Age (years)						
≤ 50	370 (52.9%)	48 (45.3%)		47 (45.2%)	48 (46.2%)	
> 50	329 (47.1%)	58 (54.7%)	0.142	57 (54.8%)	56 (53.8%)	0.889
ASA physical status						
I	30 (4.3%)	0 (0.0%)		0 (0.0%)	0 (0.0%)	
II	653 (93.4%)	101 (95.3%)		97 (93.3%)	100 (96.2%)	
III	16 (2.3%)	5 (4.7%)	**0.036**	7 (6.7%)	4 (3.8%)	0.353
HBsAg						
Negative	28 (4.0%)	5 (4.7%)		6 (5.8%)	5 (4.8%)	
Positive	671 (96.0%)	101 (95.3%)	0.731	98 (94.2%)	99 (95.2%)	0.757
Cirrhosis						
No	217 (31.0%)	28 (26.4%)		30 (28.8%)	27 (26.0%)	
Yes	482 (69.0%)	78 (73.6%)	0.334	74 (71.2%)	77 (74.0%)	0.641
Child-Pugh classification						
A	695 (99.4%)	106 (100.0%)		104 (100.0%)	104 (100.0%)	
B	4 (0.6%)	0 (0.0%)	0.435	0 (0.0%)	0 (0.0%)	–
Tumor size (cm)						
≤ 5	342 (48.9%)	40 (37.7%)		41 (39.4%)	40 (38.5%)	
> 5	357 (51.1%)	66 (62.3%)	**0.032**	63 (60.6%)	64 (61.5%)	0.887
Tumor number						
1	674 (96.4%)	100 (94.3%)		100 (96.2%)	98 (94.2%)	
> 1	25 (3.6%)	6 (5.7%)	0.299	4 (3.8%)	6 (5.8%)	0.517
Satellite nodules						
No	557 (79.7%)	82 (77.4%)		76 (73.1%)	82 (78.8%)	
Yes	142 (20.3%)	24 (22.6%)	0.581	28 (26.9%)	22 (21.2%)	0.330
Vascular invasion						
No	642 (91.8%)	93 (87.7%)		91 (87.5%)	92 (88.5%)	
Yes	57 (8.2%)	13 (12.3%)	0.162	13 (12.5%)	12 (11.5%)	0.831
Preoperative AFP (ng/ml)						
≤ 20	291 (41.6%)	60 (56.6%)		60 (57.7%)	58 (55.8%)	
20-400	170 (24.3%)	19 (17.9%)		23 (22.1%)	19 (18.3%)	
> 400	238 (34.0%)	27 (25.5%)	**0.015**	21 (20.2%)	27 (26.0%)	0.559
Preoperative ALT (units/L)						
≤ 40	386 (55.2%)	73 (68.9%)		69 (66.3%)	71 (68.3%)	
> 40	313 (44.8%)	33 (31.1%)	**0.008**	35 (33.7%)	33 (31.7%)	0.768
Preoperative AST (units/L)						
≤ 40	422 (60.4%)	67 (63.2%)		66 (63.5%)	65 (62.5%)	
> 40	277 (39.6%)	39 (36.8%)	0.577	38 (36.5%)	39 (37.5%)	0.886
Preoperative total bilirubin (μmol/L)						
≤ 17.1	529 (75.7%)	79 (74.5%)		81 (77.9%)	77 (74.0%)	
> 17.1	170 (24.3%)	27 (25.5%)	0.797	23 (22.1%)	27 (26.0%)	0.516
Preoperative direct bilirubin (μmol/L)						
≤ 6.9	582 (83.3%)	92 (86.8%)		96 (92.3%)	90 (86.5%)	
> 6.9	117 (16.7%)	14 (13.2%)	0.359	8 (7.7%)	14 (13.5%)	0.176
Preoperative albumin (g/L)						
≤ 35	34 (4.9%)	7 (6.6%)		6 (5.8%)	7 (6.7%)	
> 35	665 (95.1%)	99 (93.4%)	0.448	98 (94.2%)	97 (93.3%)	0.775
Preoperative creatinine (μmol/L)						
≤ 177	697 (99.7%)	105 (99.1%)		104 (100.0%)	103 (99.0%)	
> 177	2 (0.3%)	1 (0.9%)	0.301	0 (0.0%)	1 (1.0%)	0.316

P values of statistical significance are in bold.

ASA, American Society of Anesthesiologists; AFP, Alpha-fetoprotein; ALT, Alanine aminotransferase; AST, Aspartate aminotransferase.

### Outcomes in the overall cohort

**Figure 2:**
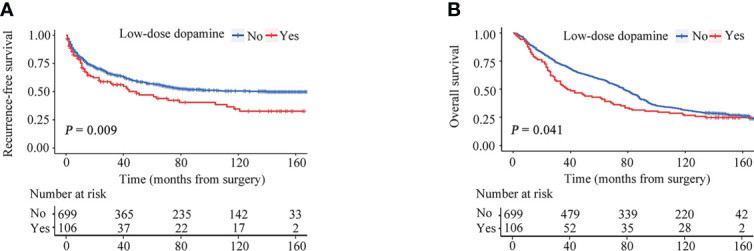
Recurrence-free survival (RFS) and overall survival (OS) curves stratified by dose of dopamine before propensity score matching. **(A)** RFS curves. **(B)** OS curves.

For the cohort as a whole, the median RFS time was 39.47 months (interquartile range [IQR], 9.60-92.73 months), and the median OS time was 74.10 months (IQR, 26.65-139.02 months). During the follow-up, tumor recurrence was observed in 307 (43.9%) patients in without dopamine group and 56 (52.8%) patients in the low-dose dopamine group. The 1-, 3-, 5- and 10-year RFS rates in the without dopamine group and the low-dose dopamine group were 78.4%, 64.1%, 56.6%, 50.4% and 70.0%, 55.9%, 46.8%, 32.4%, respectively (**Figure 2A**). The Kaplan–Meier survival curves demonstrated that patients who received low-dose dopamine use had an unfavorable RFS compared with those who did not receive dopamine (*p* = 0.009, **Figure 2A**). Regarding OS, 511 (73.1%) patients in the without dopamine group and 79 (74.5%) patients in the low-dose dopamine group had died. The 1-, 3-, 5- and 10-year OS rates in the without dopamine group and the low-dose dopamine group were 89.0%, 70.8%, 59.1%, 31.2% and 83.8%, 51.4%, 41.9%, 26.7%, respectively (**Figure 2B**). The Kaplan–Meier survival curves showed that patients with low-dose dopamine consumption had worsened OS (*p* = 0.041, **Figure 2B**). When stratified by time of recurrence, early (≤ 2 years) recurrence and late recurrence (> 2 years) were observed in 240 patients and 123 patients, respectively. Patients with low-dose dopamine use had a significantly greater chance of recurrence within 2 years (*p* = 0.025) but not after 2 years (*p* = 0.181) (**Table 2**).

**Table 2:** Recurrence in both groups by time of recurrence before propensity score matching.

	All patients	Low-dose dopamine		Log - rank
	n = 805	No (n = 699)	Yes (n = 106)	*P*-value
All recurrences, n (%)	363 (45.1%)	307 (43.9%)	56 (52.8%)	**0.009**
Time of recurrence, n (%)				
≤ 2 years	240 (29.8%)	200 (28.6%)	40 (37.7%)	**0.025**
> 2 years	123 (15.3%)	107 (15.3%)	16 (15.1%)	0.181

P values of statistical significance are in bold.

### Independent prognostic factors for RFS and OS

The predictors for RFS and OS in univariate and multivariate analyses are exhibited in **Table 3**. Univariable analysis indicated that tumor size, satellite nodules, AFP, ALT, AST, and low-dose dopamine use were associated with RFS (all *p* < 0.05), whereas Child–Pugh classification, tumor size, tumor number, satellite nodules, vascular invasion, AFP, ALT, AST, direct bilirubin, and low-dose dopamine use were associated with OS (all *p* < 0.05). Multivariate analysis indicated that satellite nodules (hazard ration [HR]: 1.483; 95% confidence interval [CI]:1.150-1.921; *p* = 0.002), AFP (HR: 1.227; 95% CI: 1.088-1.384; *p* = 0.001), low-dose dopamine (HR: 1.527; 95% CI: 1.145-2.036; *p* = 0.004) were the significant prognostic factor for RFS, and Child–Pugh classification (HR: 2.846; 95% CI: 1.029-7.869; *p* = 0.044), tumor size (HR:1.338; 95% CI: 1.127-1.588; *p* = 0.001), tumor number (HR: 1.618; 95% CI: 1.089-2.405; *p* = 0.017), satellite nodules (HR: 1.441; 95% CI: 1.183-1.755; *p* < 0.001), vascular invasion (HR: 1.482; 95% CI: 1.125-1.954; *p* = 0.005), AFP (HR: 1.114; 95% CI: 1.015-1.224; *p* = 0.023), and AST (HR: 1.230; 95% CI: 1.009-1.499; *p* = 0.040) were independent prognostic factors for OS.

**Table 3:** Univariate and multivariate analyses of recurrence-free survival and overall survival in patients before propensity score matching.

	RFS			OS		
Variables	Univariate Analysis	Multivariable Analysis		Univariate Analysis	Multivariable Analysis	
	*P*-value	HR (95% CI)	*P*-value	*P*-value	HR (95% CI)	*P*-value
Sex						
Female						
Male	0.176			0.569		
Age (years)						
≤ 50						
> 50	0.486			0.503		
ASA physical status						
I						
II						
III	0.991			0.405		
HBsAg						
Negative						
Positive	0.579			0.548		
Cirrhosis						
No						
Yes	0.051			0.438		
Child-Pugh classification						
A						
B	0.281			< 0.001	2.846 (1.029–7.869)	**0.044**
Tumor size (cm)						
≤ 5						
> 5	**0.005**	1.185 (0.957–1.467)	0.120	< 0.001	1.338 (1.127–1.588)	**0.001**
Tumor number						
1						
> 1	0.132			**0.029**	1.618 (1.089–2.405)	**0.017**
Satellite nodules						
No						
Yes	**< 0.001**	1.483 (1.150–1.912)	**0.002**	< 0.001	1.441 (1.183–1.755)	< 0.001
Vascular invasion						
No						
Yes	0.077			< 0.001	1.482 (1.125–1.954)	**0.005**
Preoperative AFP (ng/ml)						
≤ 20						
20-400						
> 400	**0.006**	1.227 (1.088–1.384)	**0.001**	**0.009**	1.114 (1.015–1.224)	**0.023**
Preoperative ALT (units/L)						
≤ 40						
> 40	**0.050**	1.119 (0.878–1.425)	0.364	**0.046**	1.049 (0.866–1.269)	0.626
Preoperative AST (units/L)						
≤ 40						
> 40	**< 0.001**	1.269 (0.990–1.626)	0.060	< 0.001	1.230 (1.009–1.499)	**0.040**
Preoperative total bilirubin (μmol/L)						
≤ 17.1						
> 17.1	0.634			0.295		
Preoperative direct bilirubin (μmol/L)						
≤ 6.9						
> 6.9	0.832			**0.014**	1.222 (0.985–1.516)	0.069
Preoperative Albumin (g/L)						
≤ 35						
> 35	0.652			0.456		
Preoperative Creatinine (μmol/L)						
≤ 177						
> 177	0.963			0.169		
Low-dose dopamine						
No						
Yes	**0.009**	1.527 (1.145–2.036)	**0.004**	**0.041**	1.260 (0.991–1.600)	0.059

P values of statistical significance are in bold.

RFS, recurrence-free survival; OS, overall survival; HR, hazard ratio; CI, confidence interval; ASA, American Society of Anesthesiologists; AFP, Alpha-fetoprotein; ALT, Alanine aminotransferase; AST, Aspartate aminotransferase.

### Recurrence and prognosis after PSM

**Figure 3:**
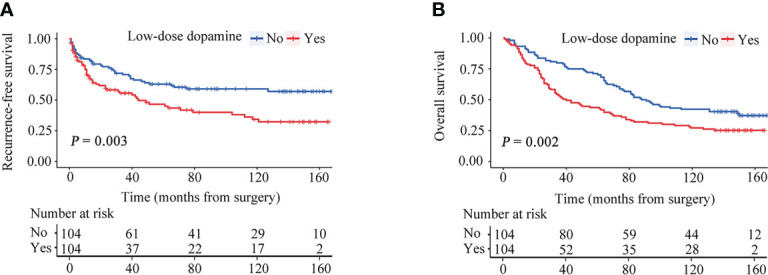
Recurrence-free survival (RFS) and overall survival (OS) curves stratified by dose of dopamine after propensity score matching. **(A)** RFS curves. **(B)** OS curves.

After PSM, the two groups were completely matched, including 208 patients. None of the baseline characteristics were significantly different (**Table 1**). Tumor recurrence was observed in 40 (38.5%) patients in the without dopamine group and 56 (53.8%) patients in the low-dose dopamine group. The 1-, 3-, 5- and 10-year RFS rates in the without dopamine group and the low-dose dopamine group were 82.4%, 70.6%, 62.9%, 59.0% and 69.5%, 55.5%, 46.4%, 34.2%, respectively (**Figure 3A**). In regard to OS, 64 (61.5%) patients in the without dopamine group and 77 (74.0%) patients in the low-dose dopamine group had died. The 1-, 3-, 5- and 10-year OS rates in the without dopamine group and the low-dose dopamine group were 93.3%, 79.8%, 71.2%, 42.3% and 84.5%, 53.4%, 43.7%, 27.2%, respectively (**Figure 3B**). Similar to the results before PSM, the low-dose dopamine-treated group still had significantly worse RFS (*p* = 0.003, **Figure 3A**) and OS (*p* = 0.002, **Figure 3B**) than the group without dopamine use in the matched cohort. In the stratified analyses, patients who infused low-dose dopamine had a significantly higher chance of recurrence within 2 years (*p* = 0.007) but not after 2 years (*p* = 0.186) (**Table 4**).

**Table 4:** Recurrence in both groups by time of recurrence after propensity score matching.

	All patients	Low-dose dopamine		Log - rank
	n = 208	No (n = 104)	Yes (n = 104)	*P*-value
All recurrences, n (%)	96 (46.2%)	40 (38.5%)	56 (53.8%)	**0.003**
Time of recurrence, n (%)				
≤ 2 years	63 (30.3%)	23 (22.1%)	40 (38.5%)	**0.007**
> 2 years	33 (15.9%)	17 (16.3%)	16 (15.4%)	0.186

P values of statistical significance are in bold.

## Discussion

This study is the first to evaluate the impact of intraoperative dopamine dosage on the long-term survival outcomes in HCC patients. We found that low-dose dopamine is an independent unfavorable prognostic risk factor in patients who underwent open radical hepatectomy. Moreover, patients with low-dose dopamine use had unsatisfactory long-term RFS and OS. These findings suggested that the administration of low-dose dopamine during hepatic surgery might be an intraoperative medication for predicting prognosis in HCC patients.

Due to the complexity of open hepatic resection, most anesthesiologists control the CVP below 5 mmHg during hepatic parenchymal transection, which significantly reduces intraoperative bleeding and provides an optimal surgical visual field (17, 18). The underlying mechanism is that the controlled low CVP accelerates venous drainage from the hepatic vein and hepatic sinusoids, which results in less backflow from the liver transected surface and less blood loss during resection. However, a lower CVP could cause renal perfusion insufficiency and reduce the effective circulating volume. Based on the pharmacological properties of dopamine, this drug may diminish the probability of low arterial perfusion due to lower intraoperative CVP. Additionally, it has been suggested that low-dose dopamine use can augment renal blood flow *via* dopaminergic receptors located on the renal vasculature (19). Previous clinical trials demonstrated that low-dose dopamine increased the renal perfusion in patients with chronic renal impairment and renovascular disease, albeit to lesser extent than in healthy people (20, 21).

Despite potent renal vasodilatation, low-dose dopamine use could not affect the liver and kidney function of HCC patients based on our present study. However, several findings were illustrated that dopamine given resulted in some side effects, such as arrhythmia and delirium. Chiolero et al. demonstrated an association between low-dose dopamine infusion and unexpected ventricular arrhythmias (22). In our study, we did not discover any cardiac complications. This fact could be ascribed to several reasons. First, Chiolero’s study selected patients with cardiac disease undergoing open-heart surgery, while our study did not enroll patients with severe cardiovascular disease. Second, hypothermia and cardioplegic solutions in cardiac surgery may have lowered the β-adrenergic stimulation threshold, which resulted in an increased incidence of arrhythmias in Chiolero’s study (22). Furthermore, Yilmaz et al. provided evidence that dopamine administration could give rise to postoperative delirium in cardiac surgical patients (23). But we did not find similar complications in our study. This difference may be attributable to higher doses of dopamine and longer use duration in Yilmaz’s study. In addition, delirium was very common after cardiac surgery (24). Therefore, further studies are required to evaluate the relationship between low-dose dopamine and clinical effects and its internal mechanism.

Despite the improvement of therapeutic strategies and surveillance plans, the postoperative recurrence rate of HCC is still high and strongly associated with poor prognosis. A series of studies have reported that AFP levels and satellite nodules are related to worse survival of liver cancer patients (25–27). These results were similar in our study. However, few studies have assessed the effect of the intraoperative dosage of dopamine on the prognosis of patients with liver cancer. In the present study, we found that the cohort assigned low-dose dopamine had worse long-term RFS and OS. These results remained similar after the use of PSM analysis to balance the confounding bias at the baseline characteristics. Several underlying molecular mechanisms by which dopamine affects prognosis may be considered. First, low-dose dopamine exerts its actions *via* the different dopamine receptor subtypes, grouped as D1-like receptors (DRD1 and DRD5) and D2-like receptors (DRD2, DRD3, and DRD4). Several studies have suggested that there is a close association between the expression of dopamine receptors and prognosis in cancer patients. We previously found that DRD1 was highly expressed in liver cancer tissues and the positive expression of DRD1 is associated with unfavorable RFS and OS in HCC patients (13). Similar results were obtained in another study, suggesting that DRD1 overexpression has a negative effect on prognosis in patients with advanced breast cancer (28). Furthermore, DRD2 agonist could suppress liver cancer cells proliferation, migration and invasion (29). To our knowledge, DRD2 has been reported higher expression in colorectal cancer and gastric cancer compared with non-tumor tissues, and DRD2 expression was related to a poor survival rate (30, 31). Second, dopamine receptor ligands might influence the biology of tumor cells and alter the tumor microenvironment in a manner independent of their behaviors on neurotransmission, which affects the function on motivation, cognition and sensory (32). Hence, growing evidence emphasizes the importance of dopamine in cancer progression.

This retrospective study has several limitations. First, we do not have information on the other factors that could affect cancer recurrence, such as perioperative opioid consumption and postoperative complications (33–35). Second, the time interval from hepatectomy to recurrence is an important prognostic factor and somewhat controversial, ranging from 6 months to 2 years after surgery (36, 37). Although we defined early recurrence based on the majority of retrospective studies, the different cut-offs may contribute to the different long-term survival outcomes in HCC patients (38, 39). Third, this study was a single-institution study. Therefore, further prospective studies are needed to validate these findings.

In conclusion, compared with no use of dopamine, intraoperatively administered low-dose dopamine has a negative impact on RFS and OS in HCC patients who undergo radical hepatectomy. Dopamine consumption may be considered a potential predictor for the prognosis of patients with HCC. The underlying mechanisms of the association between the dosage of dopamine and the long-term prognosis in HCC patients should be further investigated.

## Data availability statement

The original contributions presented in the study are included in the article/**Supplementary Material**. Further inquiries can be directed to the corresponding authors.

## Ethics statement

The studies involving human participants were reviewed and approved by the Institutional Ethics Committees of Sun Yat-sen University Cancer Center. The ethics committee waived the requirement of written informed consent for participation.

## Author contributions

All authors contributed to the study conception and design. YW, RX and YY were involved in data analysis and interpretation. YW, RX, YY and DC were involved in manuscript writing, and all the listed authors revised the submitted manuscript and approved its final version before submission.

## Funding

This work was supported by grants from the National Natural Science Foundation of China (grant 81902490 to DC and grant 82172843 to WZ), and the National Natural Science Foundation of Guangdong Province (grant 2021A1515011332 to DC and grant 2021A1515220117 to WZ), the National Key Research and Development Program of China (grant 2018YFC2001900) and Huizhou science and technology program (grant 2021 WC0106419 to YY).

## Conflict of interest

The authors declare that the research was conducted in the absence of any commercial or financial relationships that could be construed as a potential conflict of interest.

## Publisher’s note

All claims expressed in this article are solely those of the authors and do not necessarily represent those of their affiliated organizations, or those of the publisher, the editors and the reviewers. Any product that may be evaluated in this article, or claim that may be made by its manufacturer, is not guaranteed or endorsed by the publisher.
