# Supplementary material for: Intraoperative low-dose dopamine is associated with worse survival in patients with hepatocellular carcinoma: A propensity score matching analysis
Source: Front Oncol. 2022 Aug 25;12:947172. doi: 10.3389/fonc.2022.947172 (PMC9452952; doi:10.3389/fonc.2022.947172)
Supplement: Supplementary file 1 [file Table_1.docx]

Supplemental Table 1. Intraoperative and postoperative clinical characteristics of patients in without and with low-dose dopamine group

|  | Before matching | | |  | After matching | | |
| --- | --- | --- | --- | --- | --- | --- | --- |
| Characteristics | Low-dose dopamine | | *P-*value |  | Low-dose dopamine | | *P-*value |
|  | No (n=699) | Yes (n=106) |  |  | No (n=104) | Yes (n=104) |  |
| Intraoperative fluid infusion (ml) | 2872±726 | 2801±640 | 0.345 |  | 2885±583 | 2807±676 | 0.377 |
| Intraoperative urine output (ml) | 624±491 | 669±431 | 0.371 |  | 583±437 | 663±433 | 0.182 |
| Intraoperative norepinephrine use |  |  |  |  |  |  |  |
| No | 568 (81.3%) | 87 (82.1%) | 0.841 |  | 83 (79.8%) | 85 (81.7%) | 0.725 |
| Yes | 131 (18.7%) | 19 (17.9%) |  |  | 21 (20.2%) | 19 (18.3%) |  |
| Blood loss (ml) | 481±515 | 419±398 | 0.238 |  | 430±370 | 421±402 | 0.863 |
| Duration of operation (minutes) | 253±47 | 249±44 | 0.496 |  | 254±48 | 249±45 | 0.377 |
| Postoperative AFP (ng/ml) |  |  |  |  |  |  |  |
| ≤ 20 | 369 (52.8%) | 57 (53.8%) |  |  | 55 (52.9%) | 56 (53.8%) |  |
| 20-400 | 174 (24.9%) | 25 (23.6%) |  |  | 26 (25.0%) | 25 (24.0%) |  |
| > 400 | 156 (22.3%) | 24 (22.6%) | 0.958 |  | 23 (22.1%) | 23 (22.1%) | 0.986 |
| Postoperative ALT (units/L) |  |  |  |  |  |  |  |
| ≤ 40 | 4 (0.6%) | 2 (1.9%) |  |  | 0 (0.0%) | 2 (1.9%) |  |
| > 40 | 695 (99.4%) | 104 (98.1%) | 0.143 |  | 104 (100.0%) | 102 (98.1%) | 0.155 |
| Postoperative AST (units/L) |  |  |  |  |  |  |  |
| ≤ 40 | 36 (5.2%) | 5 (4.7%) |  |  | 4 (3.8%) | 5 (4.8%) |  |
| > 40 | 663 (94.8%) | 101 (95.3%) | 0.850 |  | 100 (96.2%) | 99 (95.2%) | 0.733 |
| Postoperative total bilirubin (µmol/L) |  |  |  |  |  |  |  |
| ≤ 17.1 | 237 (33.9%) | 41 (38.7%) |  |  | 35 (33.7%) | 39 (37.5%) |  |
| > 17.1 | 462 (66.1%) | 65 (61.3%) | 0.335 |  | 69 (66.3%) | 65 (62.5%) | 0.562 |
| Postoperative direct bilirubin (µmol/L) |  |  |  |  |  |  |  |
| ≤ 6.9 | 209 (29.9%) | 37 (34.9%) |  |  | 29 (27.9%) | 35 (33.7%) |  |
| > 6.9 | 490 (70.1%) | 69 (65.1%) | 0.297 |  | 75 (72.1%) | 69 (66.3%) | 0.367 |
| Postoperative creatinine  (µmol/L) |  |  |  |  |  |  |  |
| ≤ 177 | 698 (99.9%) | 105 (99.1%) |  |  | 104 (100.0%) | 103 (99.0%) |  |
| > 177 | 1 (0.1%) | 1 (0.9%) | 0.123 |  | 0 (0.0%) | 1 (1.0%) | 0.316 |

Abbreviations: AFP: Alpha-fetoprotein; ALT: Alanine aminotransferase; AST: Aspartate aminotransferase.
